# Surfactant effects on the viability and function of human mesenchymal stem cells: in vitro and in vivo assessment

**DOI:** 10.1186/s13287-017-0634-y

**Published:** 2017-08-03

**Authors:** Chung-Ming Chen, Hsiu-Chu Chou, Willie Lin, Chris Tseng

**Affiliations:** 1 Department of Pediatrics, Taipei Medical University Hospital, Taipei, Taiwan; 2 Department of Pediatrics, School of Medicine, College of Medicine, Taipei Medical University, Taipei, Taiwan; 3 Department of Anatomy and Cell Biology, School of Medicine, College of Medicine, Taipei Medical University, Taipei, Taiwan; 4 Meridigen Biotech Co., Ltd., Taipei, Taiwan

**Keywords:** Surfactant, Mesenchymal stem cells, Hyperoxia, Mean linear intercept, Alveolarization

## Abstract

**Background:**

Surfactant therapy has become the standard of care for preterm infants with respiratory distress syndrome. Preclinical studies have reported the therapeutic benefits of mesenchymal stem cells (MSCs) in experimental bronchopulmonary dysplasia. This study investigated the effects of a surfactant on the in vitro viability and in vivo function of human MSCs.

**Methods:**

The viability, phenotype, and mitochondrial membrane potential (MMP) of MSCs were assessed through flow cytometry. The in vivo function was assessed after intratracheal injection of human MSCs (1 × 10^5^ cells) diluted in 30 μl of normal saline (NS), 10 μl of a surfactant diluted in 20 μl of NS, and 10 μl of a surfactant and MSCs (1 × 10^5^ cells) diluted in 20 μl of NS in newborn rats on postnatal day 5. The pups were reared in room air (RA) or an oxygen-enriched atmosphere (85% O_2_) from postnatal days 1 to 14; eight study groups were examined: RA + NS, RA + MSCs, RA + surfactant, RA + surfactant + MSCs, O_2_ + NS, O_2_ + MSCs, O_2_ + surfactant, and O_2_ + surfactant + MSCs. The lungs were excised for histological and cytokine analysis on postnatal day 14.

**Results:**

Compared with the controls, surfactant-treated MSCs showed significantly reduced viability and MMP after exposure to 1:1 and 1:2 of surfactant:MSCs for 15 and 60 minutes. All human MSC samples exhibited similar percentages of CD markers, regardless of surfactant exposure. The rats reared in hyperoxia and treated with NS exhibited a significantly higher mean linear intercept (MLI) than did those reared in RA and treated with NS, MSCs, surfactant, or surfactant + MSCs. Treatment with MSCs, surfactant, or surfactant + MSCs significantly reduced the hyperoxia-induced increase in MLI. The O_2_ + surfactant + MSCs group exhibited a significantly higher MLI than did the O_2_ + MSCs group. Furthermore, treatment with MSCs and MSCs + surfactant significantly reduced the hyperoxia-induced increase in apoptotic cells.

**Conclusions:**

Combination therapy involving a surfactant and MSCs does not exert additive effects on lung development in hyperoxia-induced lung injury.

**Electronic supplementary material:**

The online version of this article (doi:10.1186/s13287-017-0634-y) contains supplementary material, which is available to authorized users.

## Background

Respiratory distress syndrome (RDS) is a major cause of morbidity and mortality in preterm infants. Despite early surfactant therapy, optimal ventilation strategies, and increased use of noninvasive positive pressure ventilation, bronchopulmonary dysplasia (BPD) remains a complication in premature infants with RDS [1]. Many infants with BPD experience significant respiratory morbidity, including increased airway reactivity and obstructive airway disease, throughout childhood [2]. Some abnormal lung functions may persist into adulthood [3].

Surfactant therapy has become the standard of care for preterm infants with RDS and was reported to reduce the combined outcomes of death and BPD in such patients [4]. A recent meta-analysis concluded that compared with delayed surfactant treatment, early surfactant treatment significantly reduced mortality, air leak, BPD, and BPD or death [5]. Mesenchymal stem cells (MSCs) are multipotent stromal cells that self-renew and differentiate into various cell types, including bone, cartilage, adipose tissue, muscle, and tendon cells [6]. MSCs have immunomodulatory, anti-inflammatory, and regenerative effects [7]. Preclinical studies have reported the therapeutic benefits of bone marrow- and cord blood-derived MSCs in chronic oxygen-induced lung injury in rodents [8–19]. The marked advancement in animal models has stimulated the use of this treatment in clinical settings. A phase I clinical trial successfully demonstrated the safety and feasibility of transplanting allogeneic human umbilical cord blood-derived MSCs in preterm infants [20]. An animal study revealed that the early (at P3), rather than late (at P10), intratracheal transplantation of MSCs improved hyperoxia-induced lung injury in newborn rats [21]. However, the optimal timing of MSCs transplantation after surfactant administration remains unestablished.

McDonald et al. [22] observed that the addition of a surfactant (Curosurf) does not alter the viability and function of human amniotic epithelial cells and suggested that a combination therapy of these epithelial cells and a surfactant is an effective therapy for ameliorating preterm lung disease. However, the effects of other surfactants on the viability and in vivo function of human MSCs remain unknown. In this study, we hypothesized that exogenous surfactant instillation improves the development of the lung and that the surfactant plus human MSCs combination enhances the effects of MSCs on experimental BPD in rats. This study investigated the effects of an animal-derived surfactant (Survanta) on the in vitro viability and phenotype and in vivo function of human MSCs. Furthermore, the study identified the additive effects of a surfactant and human MSCs on hyperoxia-induced lung injury in neonatal rats.

## Methods

### Isolation of human MSCs and surfactant treatment

MSCs were isolated from human placenta, as described previously [23]. Written informed consent was obtained from individual mothers before the study and the study was approved by the Ethics Committee of the Tri-Service General Hospital. Briefly, the cells were isolated and maintained in α-minimum essential medium, supplemented with 10% fetal bovine serum (FBS), 2 mM L-glutamine, and 1 ng/ml basic fibroblast growth factor at 37 °C under saturated humidity and 5% CO_2_. The MSCs were characterized by analyzing the expression of CD markers (CD44, CD73, CD90, CD105, CD11b, CD19, CD34, and CD45) and the human leukocyte antigen-antigen D-related complex through flow cytometry (BD Stemflow^™^ hMSC Analysis Kit, BD, Franklin Lakes, NJ, USA). The capability of trilineage differentiation (osteocytes, chondrocytes, and adipocytes) and the karyotyping result were examined, which revealed positive results. The MSCs, in a cryovial, were thawed in a 37 °C water bath for 2 minutes, the cells (1 × 10^7^ cells/ml) were cocultured with surfactant + MSCs at a ratio of 1:1, 1:2, and 1:8 at 37 °C under 5% CO_2_ for 15 and 60 minutes.

### Detection of cell viability

The cell viability was assessed through flow cytometry using the annexin V-FITC Apoptosis Detection Kit (BD Biosciences, San Diego, CA, USA). Briefly, human MSCs were grown in 24-well plates and treated with a surfactant for 15 or 60 minutes. The cells were harvested and washed twice in cold staining buffer [2% FBS in phosphate-buffered saline (PBS)]and resuspended in 1 × binding buffer at a concentration of 1 × 10^6^ cells/ml. Subsequently, the cells were stained with annexin V and propidium iodide (BD Biosciences). The cell death was analyzed through flow cytometry by using a FACSCanto II flow cytometer (BD Biosciences) within 1 hour.

### Assessment of mitochondrial membrane potential

The MMP of the treated MSCs was evaluated using the MitoProbe JC-1 Assay Kit (M34152, Molecular Probes, Grand Island, NY, USA), as instructed by the manufacturer, and quantified through flow cytometry. Briefly, the cells were incubated with a lipophilic cationic dye, JC-1 (2 μM, 5,6,6′-tetrachloro-1,1′,3,3′-tetraethylbenzimidazolylcarbocyanine iodide), and an MMP disrupter, carbonyl cyanide 3-chlorophenylhydrazone (50 M), was used as a positive control. The samples were analyzed using an EPICS XL flow cytometer (Beckman Coulter Electronics Ltd., Fullerton, CA, USA). JC-1 monomers and aggregates were detected in the FL1 and FL2 channels, respectively. Aggregated, mitochondrially imported JC-1 emits orange fluorescence (FL2, 590 nm), whereas unaggregated JC-1 emits green fluorescence (FL1, 520 nm).

### Phenotypic analysis of MSCs through flow cytometry

CD markers of the surfactant-treated MSCs were analyzed according to method recommended by the International Society of Cell Therapy. Single-color flow cytometry was performed by staining 1 × 10^6^ cells with a primary antibody for 30 minutes on ice. The relevant isotype control antibody was used as a negative control. The cells were subsequently washed with fluorescence-activated cell sorting (FACS) buffer (2% FBS in PBS) and centrifuged at 300 *g* for 5 minutes at 4 °C. Data were acquired using the BD FACSCanto II flow cytometer and were analyzed using Flowlogic software (FlowJo, Ashland, OR, USA). All primary antibodies were purchased from BD Biosciences, North Ryde, NSW, Australia.

### In vivo effects of the surfactant on the function of human MSCs

#### Transplantation of human MSCs

**Fig. 1:**
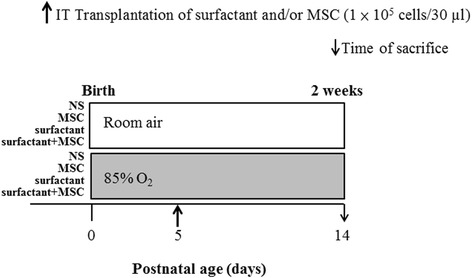
Diagrammatic representation of the experimental design showing the study timeline and the treatment groups. *MSCs* mesenchymal stem cells, *NS* normal saline

Our study was approved by the Animal Care and Use Committee at Taipei Medical University. Time-dated pregnant Sprague-Dawley rats were housed in individual cages with 12-h light-dark cycles. Within 12 hours of birth, litters were pooled and randomly redistributed to the newly delivered mothers, and the pups were then randomly assigned to room air (RA) or oxygen-enriched atmosphere (O_2_) treatment. The pups in O_2_ treatment subgroups were reared in an atmosphere containing 85% O_2_ from postnatal days 1 to 14. The pups in RA control subgroups were reared in normal RA for 14 days. To avoid oxygen toxicity in the nursing mothers, they were rotated between the O_2_ treatment and RA control litters every 24 hours. An oxygen-rich atmosphere was maintained in a transparent 40 × 50 × 60-cm plexiglass chamber receiving O_2_ continuously at 4 L/min. Oxygen levels were monitored using a ProOx P110 monitor (Bio-Spherix; Redfield, NY, USA). For intratracheal administration, the rat pups were anesthetized with 2% isoflurane (Halocarbon Laboratories, River Edge, NJ, USA), positioned against an angled restraining stand, and exposed to locate the trachea. Human MSCs (1 × 10^5^ cells) in 30 μl of normal saline (NS); 10 μl of a surfactant (Survanta, Abbvie, North Chicago, IL, USA), corresponding to approximately 35 mg/kg of phospholipids, diluted in 20 μl of NS; and 10 μl of the surfactant and MSCs (1 × 10^5^ cells) diluted in 20 μl of NS were administered into the rat trachea by using a 30-gauge syringe (Hamilton Company, Reno, NY, USA) on postnatal day 5 (Fig. 1). We held the syringe upright and slowly injected the solution into the lungs during inspiratory phase. We examined eight study groups: RA + NS, RA + MSCs (1 × 10^5^ cells), RA + surfactant, RA + surfactant + MSCs (1 × 10^5^ cells), O_2_ + NS, O_2_ + MSCs (1 × 10^5^ cells), O_2_ + surfactant, and O_2_ + surfactant + MSCs (1 × 10^5^ cells). The surfactant + MSCs mixture was administered within 10 minutes of combination. Thereafter, the rats were allowed to recover from anesthesia and were returned to their mothers. The rats from each group were strongly anesthetized with an overdose of isoflurane on postnatal day 14, and body and lung weights were recorded. Immediately after death, the left lung was ligated and the right lung was fixed by tracheal instillation of 10% buffered formalin at a pressure of 25 cm H_2_O for 10 minutes.

#### Cytokine levels

Lung tissues (0.06 g) were homogenized in 0.6 ml of ice-cold lysis buffer containing 1% Nonidet P-40, 0.1% SDS, 0.01 M deoxycholic acid and a complete protease cocktail inhibitor. The samples were then centrifuged at 13,000 rpm for 20 minutes at 4 °C and the supernatant were aliquoted and stored at -20 °C. Proteins (30 μg) were resolved in 8 ~ 15% SDS-PAGE in reduced conditions and electroblotted to a polyvinylidene difluoride membrane, ImmobilonP (Millipore, IPVH00010, Bedford, MA, USA). After blocking with 5% nonfat milk, the membranes were incubated with anti-interleukin (IL)-1β (GTX55675, GeneTex, San Antonio, TX, USA) and anti-macrophage inflammatory protein (MIP)-2 (JJ19, Santa Cruz Biotechnology, Dallas, TX, USA) at 4 °C overnight. Mouse anti-β-actin mAb (C4, 1:1000; Santa Cruz Biotechnology) was used as an internal control.

#### Lung histology

To standardize the analysis, tissue sections were obtained from the right middle lobe of the right lung. Five-micron lung tissue sections were stained with hematoxylin and eosin, examined through light microscopy, and assessed for lung morphometry. The mean linear intercept (MLI), an indicator of the mean alveolar diameter, was assessed in 10 nonoverlapping fields [23]. A terminal deoxynucleotidyl transferase dUTP nick-end labeling (TUNEL) assay was performed to detect apoptotic DNA damage by using the APO-BRDU-IHC In Situ DNA Fragmentation Assay Kit (BioVision, Mountain View, CA, USA). Positively stained apoptotic cells in the lung were counted randomly in ten fields (×400) [24].

#### Statistical analysis

Data are presented as the means ± standard deviation. Differences among the groups were analyzed using one-way analysis of variance, and the significance was determined using Bonferroni’s correction for multiple comparisons. The survival rate was evaluated using the Kaplan–Meier method, and the log-rank test was used for intergroup comparisons. Differences were considered significant if *P* < 0.05.

## Results

### Surfactant reduces the viability of human MSCs

**Fig. 2:**
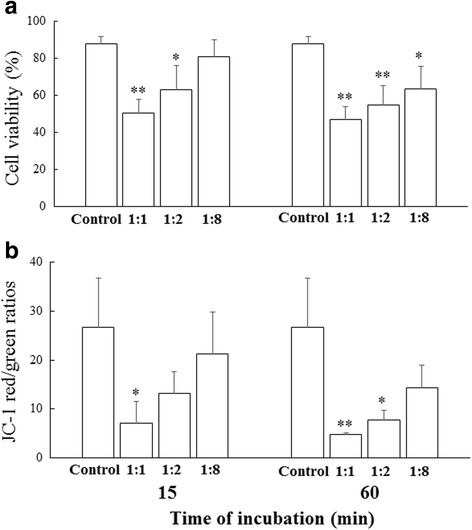
Viability and MMP of human MSCs after 15 and 60 minutes of exposure to varying surfactant concentrations. The surfactant was cocultured with human MSCs at a ratio of 1:1, 1:2, and 1:8 for 15 and 60 minutes. The cell viability was assessed through flow cytometry (**a**), and the MMP was assessed using the JC-1 *red:green* fluorescence ratio (**b**). The FACS quantitative analysis of changes in the mitochondrial potential of the inner membrane of MSCs. The results are presented as ratios of *red:green* fluorescence signals from each sample, indicating mitochondrial metabolism following the incubation of the cells with the surfactant, compared with the controls. The results are presented as the mean of the experiments performed in triplicate ± SD. *P < 0.05 and **P < 0.01 vs. controls

Compared with the controls, the surfactant-treated MSCs showed significantly reduced viability after exposure to 1:1 and 1:2 of surfactant:MSC for 15 minutes. Furthermore, the cell viability of the surfactant-treated MSCs decreased significantly after exposure to 1:1, 1:2, and 1:8 of surfactant:MSC for 60 minutes, compared with the controls (Fig. 2a).

### Surfactant induces mitochondrial dysfunction in human MSCs

To assess the mitochondrial membrane potential (MMP), we stained the MSCs with JC-1, which aggregates in the inner mitochondrial membrane to exhibit red and green fluorescence in normal polarized and depolarized mitochondrial membranes, respectively [25]. We assessed the ratio of the red (aggregated) and green (monomeric) forms of JC-1 in the surfactant-treated MSCs. This analysis revealed a significant decrease in the JC-1 red:green ratio of the cells exposed to 1:1 of surfactant:MSC compared with the controls after 15 minutes and a significant decrease in the MMP of the cells exposed to 1:1 and 1:2 of surfactant:MSC compared with the controls after 60 minutes (Fig. 2b).

### Expression of cell surface markers on surfactant-treated human MSCs

#### Characterization of human MSCs through FACS

**Fig. 3:**
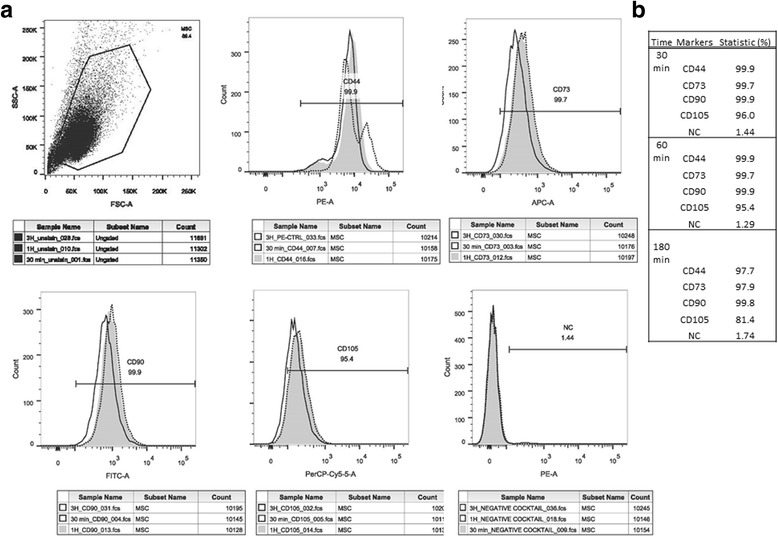
Representative flow cytometry data to characterize phenotypes of human MSCs. **a** The cells were stained with fluorochrome-conjugated antibodies against CD44, CD73, CD90, CD105, and control IgG. The plots show the staining profile of an isotype control IgG against that of a specific antibody. **b** The percentage of positive cells for each marker remained constant after the surfactant treatment. *MSCs* mesenchymal stem cells

The surface marker expression profile of human MSCs was examined through flow cytometry; Fig. 3 presents the percentage of cells positive for each marker. Phenotypically, the MSCs were positive for CD44, CD73, CD90, and CD105, resembling a typical marker expression pattern (Fig. 3a). The surface markers of human MSCs and percentage of cells positive for each marker remained constant after the surfactant treatment (Fig. 3b). These results indicate that the surfactant did not alter the phenotype of human MSCs in this study.

#### Survival

**Fig. 4:**
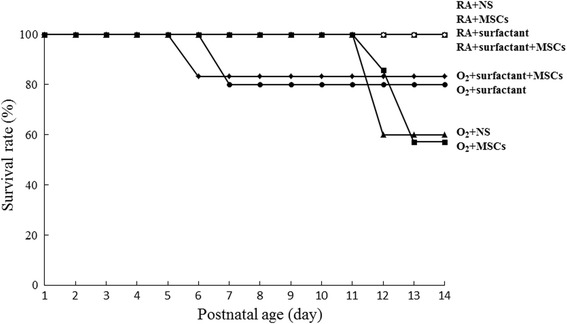
Effects of surfactant and human MSCs on the survival rate on postnatal day 14. *MSCs* mesenchymal stem cells, *NS* normal saline, *O*_*2*_ oxygen-enriched atmosphere, *RA* room air

In this study, six dams gave birth to 56 pups. Of the 31 RA-exposed pups, six, ten, eight, and seven were treated with NS, MSCs, surfactant, and surfactant + MSCs, respectively. Of the 25 hyperoxia-exposed rats, five, seven, six, and seven were treated with NS, MSCs, surfactant, and surfactant + MSCs, respectively. The rats who received the aforementioned treatments in RA survived (Fig. 4). The rats who were reared in hyperoxia and received the surfactant or MSCs + surfactant exhibited a higher survival rate than did those who were reared in hyperoxia and received NS or MSCs. The differences in the survival rate of the NS-, MSC-, or surfactant-treated groups were not statistically significant.

#### Body and lung weights and lung:body weight ratio

The rats who received the treatments in hyperoxia exhibited significantly lower body weights on postnatal day 14 than did those who received the treatments in RA (Table 1). The lung weight was significantly lower in the rats reared in hyperoxia and treated with surfactant + MSCs than in those reared in RA and treated with MSCs, surfactant, or surfactant + MSCs. However, the lung:body weight ratios were comparable in the rats reared in RA and hyperoxia.

**Table 1:** Body and lung weights and lung:body weight ratios in 14-day-old rats exposed to RA or hyperoxia and treated with NS, MSCs, surfactant, or MSCs + surfactant on postnatal day 5

Treatment	*n*	Body weight (g)	Lung weight (g)	Lung:body weight (%)
RA + NS	6	22.23 ± 2.21	0.33 ± 0.02	1.48 ± 0.17
RA + MSCs	10	22.33 ± 2.01	0.34 ± 0.02	1.48 ± 0.11
RA + surfactant	8	23.47 ± 1.28	0.35 ± 0.03	1.50 ± 0.09
RA + surfactant + MSCs	7	23.38 ± 1.48	0.35 ± 0.03	1.50 ± 0.08
O_2_ + NS	3	16.42 ± 3.79**	0.28 ± 0.08	1.66 ± 0.23
O_2_ + MSCs	4	16.38 ± 3.89**	0.27 ± 0.09	1.62 ± 0.13
O_2_ + surfactant	4	16.48 ± 2.64**	0.26 ± 0.05	1.57 ± 0.13
O_2_ + surfactant + MSCs	5	15.13 ± 5.13**	0.24 ± 0.08**	1.56 ± 0.18

Values are presented as mean ± standard deviation

*MSCs* mesenchymal stem cells, *NS* normal saline, *O*_*2*_ oxygen-enriched atmosphere, *RA* room air

**P < 0.01 vs. RA + MSCs, RA + surfactant, and RA + surfactant + MSCs

#### Cytokine levels

**Fig. 5:**
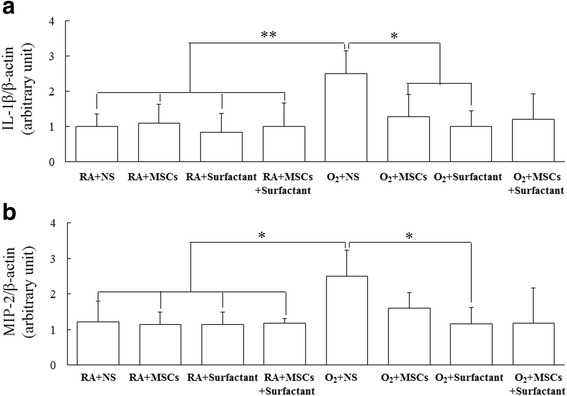
**a** IL-1β and (**b**) MIP-2 levels in lung tissues of 14-day-old rats exposed to RA or hyperoxia and treated with NS, MSCs, surfactant, or MSCs + surfactant on postnatal day 5. The rats reared in hyperoxia and treated with NS exhibited significantly higher IL-1β and MIP-2 than did those reared in RA and treated with NS, MSCs, or surfactant. The O_2_ + MSCs and O_2_ + MSCs + surfactant groups and the O_2_ + surfactant group exhibited a significantly lower IL-1β and lower MIP-2 than did the O_2_ + NS group, respectively. *P < 0.05. *IL* interleukin, *MIP* macrophage inflammatory protein, *MSCs* mesenchymal stem cells, *NS* normal saline, *O*_*2*_ oxygen-enriched atmosphere, *RA* room air

The rats reared in hyperoxia and treated with NS exhibited significantly higher IL-1ß and MIP-2 than did those reared in RA and treated with NS, MSCs, or surfactant (Fig. 5). Treatment with MSCs or surfactant reduced the hyperoxia-induced increase in the IL-1β and MIP-2. The O_2_ + MSCs and O_2_ + MSCs + surfactant groups and the O_2_ + surfactant group exhibited a significantly lower IL-1ß and lower MIP-2 than did the O_2_ + NS group, respectively.

#### Histological results

**Fig. 6:**
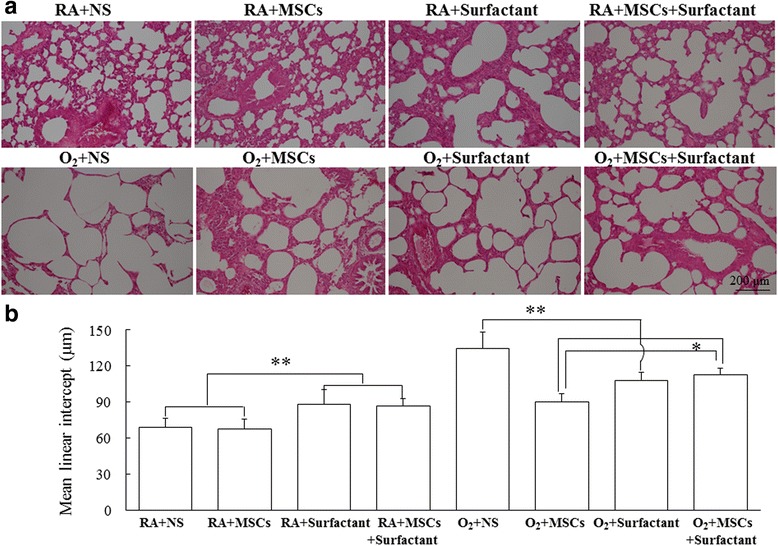
**a** Representative histology and (**b**) MLI in 14-day-old rats exposed to RA or hyperoxia and treated with NS, MSCs, surfactant, or MSCs + surfactant on postnatal day 5. The rats reared in hyperoxia and treated with NS exhibited a significantly higher MLI than did those reared in RA and treated with NS, MSCs, or surfactant. Treatment with MSCs or surfactant significantly reduced the hyperoxia-induced increase in the MLI. The O_2_ + MSCs + surfactant group exhibited a significantly higher MLI than did the O_2_ + MSCs group.*P < 0.05 and **P < 0.01. *MSCs* mesenchymal stem cells, *NS* normal saline, *O*_*2*_ oxygen-enriched atmosphere, *RA* room air

Figure 6a shows lung tissue sections stained with hematoxylin and eosin on postnatal day 14. The rats reared in hyperoxia and treated with NS yielded a significantly higher MLI than did those reared in RA and treated with NS, MSCs, surfactant, or surfactant + MSCs (Fig. 6b). Treatment with MSCs, surfactant, or surfactant + MSCs significantly reduced the hyperoxia-induced increase in the MLI. The O_2_ + surfactant + MSCs group exhibited a significantly higher MLI than did the O_2_ + MSCs group. The rats reared in RA and treated with the surfactant or surfactant + MSCs showed a significantly increased MLI compared with those treated with NS or MSCs. We performed immunohistochemistry by antihuman nuclear antibody and found hMSCs in the MSCs-treated lung sections (Additional file 1: Figure S1).

**Fig. 7:**
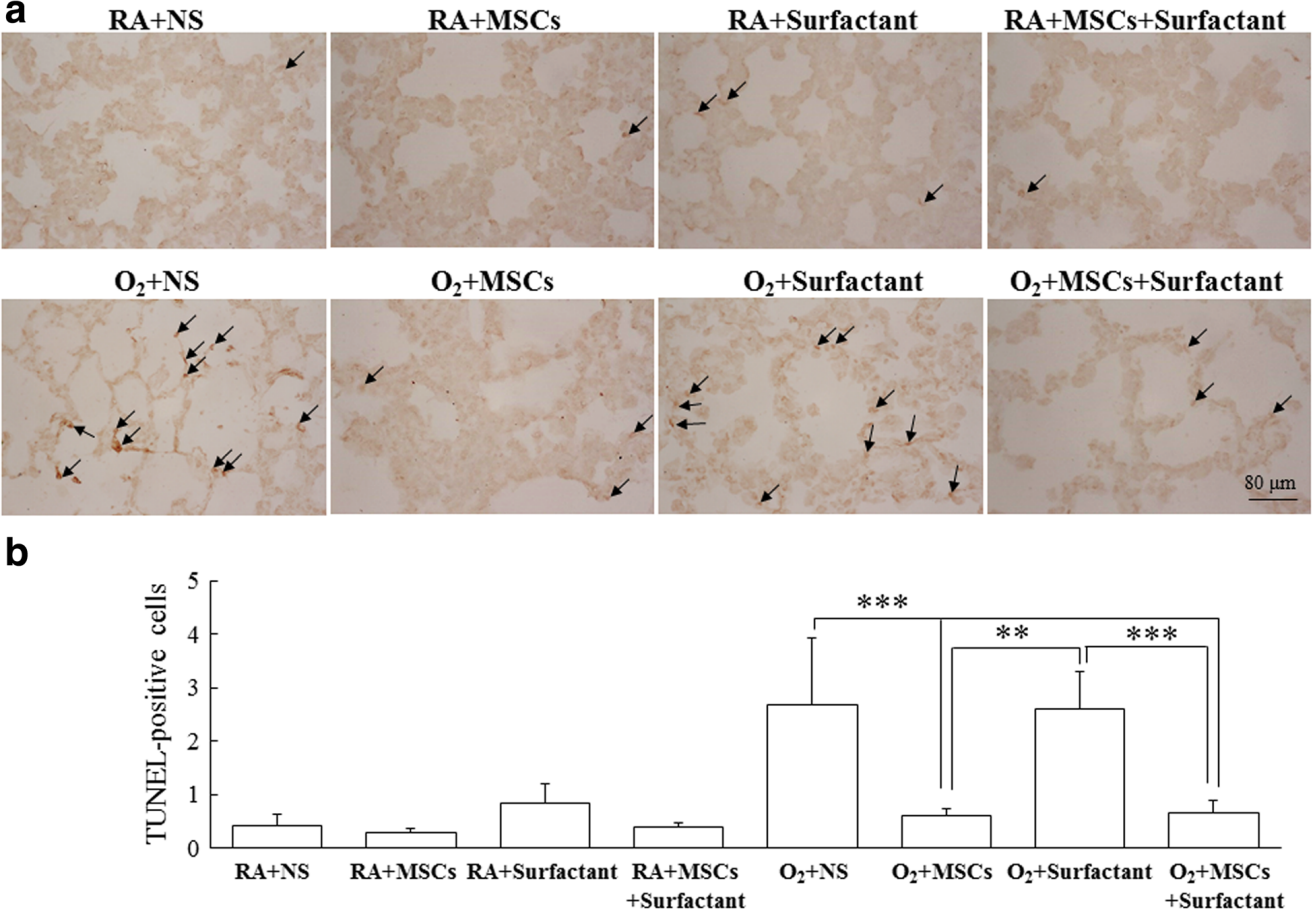
**a** Representative TUNEL staining of the lung tissue sections and (**b**) semi-quantitative measurement of apoptosis by counting TUNEL-positive cells. Positive staining is indicated in *brown* (*arrow*). The rats reared in hyperoxia and treated with NS or the surfactant exhibited significantly higher TUNEL-positive cells than did those reared in RA. Treatment with MSCs or MSCs + surfactant significantly reduced the hyperoxia-induced increase in TUNEL-positive cells. **P < 0.01 and ***P < 0.001. *MSCs* mesenchymal stem cells, *NS* normal saline, *O*_*2*_ oxygen-enriched atmosphere, *RA* room air

Figure 7a shows lung tissue sections stained with TUNEL reagents for assessing apoptosis. The rats reared in hyperoxia and treated with NS or the surfactant showed significantly higher TUNEL-positive cells than did those reared in RA (Fig. 7b). Treatment with MSCs or surfactant + MSCs significantly reduced the hyperoxia-induced increase in TUNEL-positive cells.

## Discussion

In our in vitro model, the surfactant treatment for 15 and 60 minutes reduced the viability and MMP of human MSCs. Neonatal hyperoxia exposure arrested alveolarization in the lungs of rat offspring on postnatal day 14. The intratracheal administration of MSCs, surfactant, or surfactant + MSCs on postnatal day 5 improved alveolarization in hyperoxia-exposed neonatal rats. The major findings are that the surfactant reduced the in vitro viability and in vivo function of human MSCs through mitochondrial dysfunction. The intratracheal administration of surfactant + MSCs did not enhance the effects of the MSCs on alveolarization in hyperoxia-induced lung injury in neonatal rats.

Our study demonstrated that on postnatal day 14, the rats reared in hyperoxia had lower body weights than did those reared in RA and treated with MSCs, surfactant, or surfactant + MSCs. These reduction effects of hyperoxia on body weights are consistent with our previous study [26]. Body weights were comparable among the rats reared in hyperoxia and treated with NS, MSCs, surfactant, or surfactant + MSCs. These results suggest that the body weight was mainly influenced by hyperoxia and not by MSC or surfactant treatment; lung growth was arrested during a 2-week exposure to 85% O_2_.

Mitochondria play critical roles in cell survival. Mitochondrial outer membrane permeabilization and the release of cytochrome c and other proapoptotic particles are early signaling actions that activate the apoptotic pathway. To determine whether a surfactant induces apoptosis of the MSCs by inducing mitochondrial outer membrane permeabilization, we stained the treated MSCs with JC-1 and subjected them to flow cytometry. The dual-fluorescent dye, JC-1, accumulates in mitochondria and functions as a sensor of MMP, which is an important marker of mitochondrial health that declines rapidly during mitochondrial outer membrane permeabilization. This study revealed a significant decrease in the JC-1 red:green ratio (decreased MMP) of the MSCs exposed to 1:1 and 1:2 of surfactant: MSC compared with the controls after 15 and 60 minutes. These results indicate that the surfactant reduced the in vitro viability of human MSCs through mitochondrial dysfunction.

Alveolarization is an intricate event that is affected by numerous insults, including infections, inspired oxygen concentration, and mechanical ventilation [27]. The cytokine concentration was increased in the amniotic fluid and tracheal aspirate of newborns with BPD [28]. Oxygen therapy in newborn infants with RDS increases oxidative stress and results in cytokine production [29]. The role of cytokines in BPD has been supported by human and animal studies reporting that increased cytokine concentrations and inflammatory cells are associated with BPD development [30, 31]. In this study, we found that rats reared in hyperoxia and treated with NS exhibited significantly higher IL-1ß and MIP-2 than did those reared in RA and MSCs or surfactant treatments reduced the cytokine levels. We measured the MLI as a parameter of alveolarization and observed that compared with RA exposure, hyperoxia exposure significantly increased the MLI and that MSCs and surfactant treatments reduced the MLI. MSCs have immunomodulatory and anti-inflammatory effects and have been reported to reduce maternal inflammation and neonatal hyperoxia-induced increase in tumor necrosis factor-α and interleukin-6 levels [7, 23]. We speculated that the therapeutic effects of MSCs on the development of the lung are mediated through the inhibition of proinflammatory cytokine production.

Pulmonary surfactants increase lung compliance by reducing the surface tension of water. In this study, the rats reared in RA and treated with the surfactant or surfactant + MSCs showed a significantly higher MLI than did those reared in RA and treated with NS or MSCs. A larger MLI represents more pronounced airspace enlargement. These findings are supported by Cattarossi et al. [32] who reported an increased alveolar radius and lung compliance after intratracheal surfactant instillation by using a stereomicroscope in preterm rabbits. Apoptotic cells in the lungs were evaluated through TUNEL staining. In the present study, the MSCs exerted favorable effects on apoptosis, whereas the surfactant did not prevent apoptosis in hyperoxia-induced lung injury.

A surfactant is composed of lipids and several specific surfactant proteins, which have a unique spreading property and can reduce the surface tension. The intratracheal administration of surfactant + budesonide compared with that of a surfactant alone significantly reduced the incidence of BPD or death in very low birth weight infants with severe RDS [33]. These characteristics make a surfactant an ideal carrier to instill MSCs into the lung. In this study, neonatal hyperoxia exposure arrested alveolarization in the lungs of rat offspring on postnatal day 14. The intratracheal administration of MSCs, surfactant, or surfactant + MSCs on postnatal day 5 improved alveolarization. The administration of surfactant + MSCs reduced alveolarization compared with that of MSCs alone. These results do not support our hypothesis that surfactant plus human MSCs combination enhances the effects of MSCs on alveolarization in hyperoxia-induced lung injury in neonatal rats. Additional studies are necessary to determine the optimal timing of stem cell transplantation in surfactant-treated infants.

## Conclusions

The surfactant reduced the in vitro viability of human MSCs through mitochondrial dysfunction. It also reduced the in vivo protective effects of MSCs on alveolarization in hyperoxia-exposed neonatal rats. These results indicate that the combination therapy of a surfactant and MSCs does not have additive effects on lung development in hyperoxia-induced lung injury.

## Additional file

Additional file 1: Figure S1. Representative immunohistochemical staining of lung sections with anti-human nuclei antibody. (TIF 5123 kb)

## Abbreviations

BPD: Bronchopulmonary dysplasia
FACS: Fluorescence-activated cell sorting
FBS: Fetal bovine serum
IL: Interleukin
MIP: Macrophage inflammatory protein
MLI: Mean linear intercept
MMP: Mitochondrial membrane potential
MSCs: Mesenchymal stem cells
NS: Normal saline
O_2_: Oxygen-enriched atmosphere
PBS: Phosphate-buffered saline
RA: Room air
RDS: Respiratory distress syndrome
TUNEL: Terminal deoxynucleotidyl transferase dUTP nick-end labeling
