# Supplementary figures and images for: Surfactant effects on the viability and function of human mesenchymal stem cells: in vitro and in vivo assessment
Source: Stem Cell Res Ther. 2017 Aug 3;8:180. doi: 10.1186/s13287-017-0634-y (PMC5543543; doi:10.1186/s13287-017-0634-y)

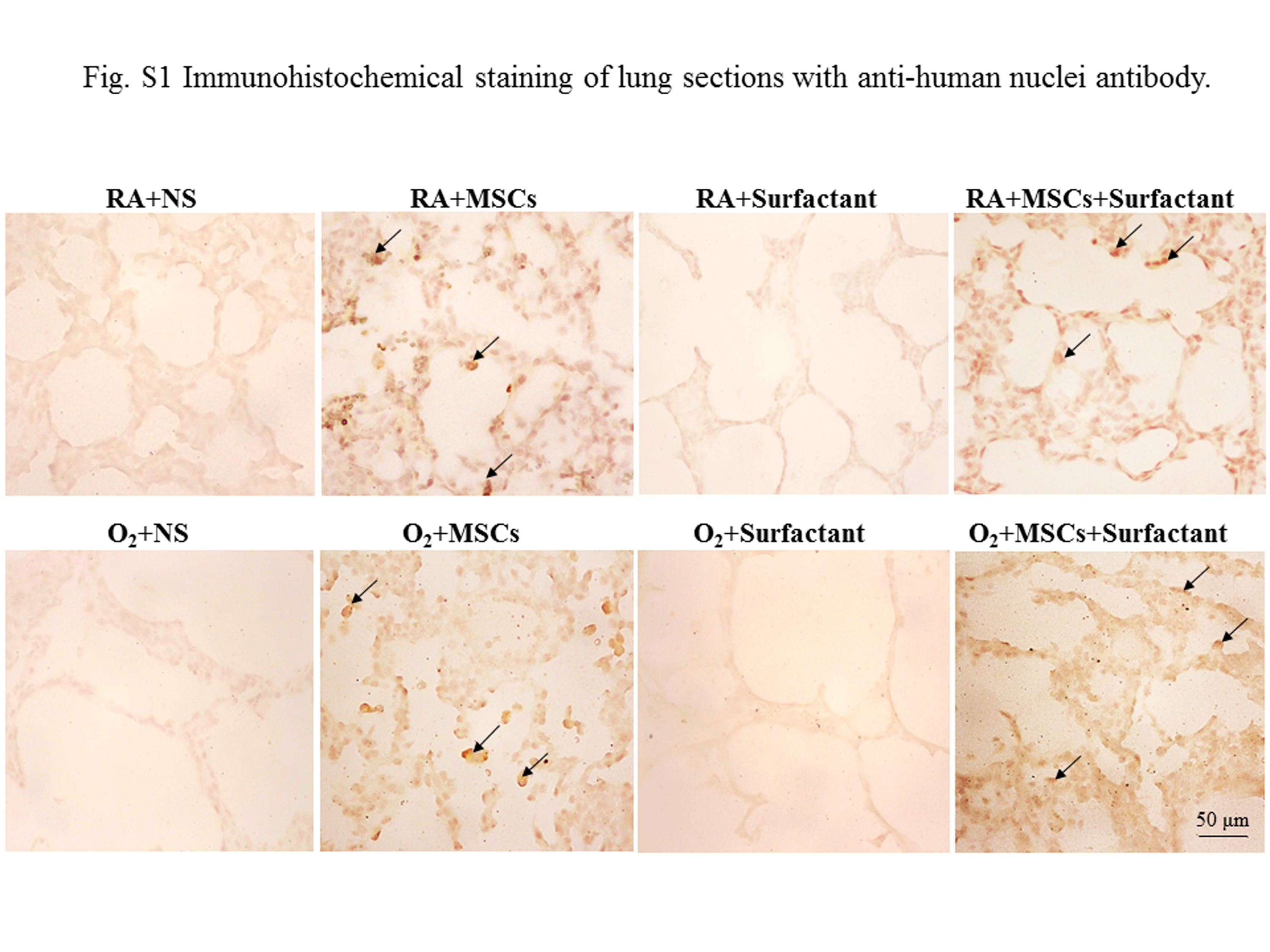

Supplement: Additional file 1: Figure S1. — Representative immunohistochemical staining of lung sections with anti-human nuclei antibody. (TIF 5123 kb) [file 13287_2017_634_MOESM1_ESM.tif]
